# Biliary Ascariasis After Worm Removal from the Duodenum and Single-Dose Albendazole Treatment

**DOI:** 10.4269/ajtmh.2010.09-0793

**Published:** 2010-07

**Authors:** Arthit Wongsaensook, Wattana Sukeepaisarnjaroen, Kittisak Sawanyawisuth

**Affiliations:** Department of Medicine, Faculty of Medicine, Srinagarind Hospital, Khon Kaen University, Khon Kaen, Thailand

A 46-year-old woman, after having a cholecystectomy, presented with severe vomiting and abdominal pain for 2 days. Physical examination revealed afebrile, no jaundice, moderate abdominal distention, hyperactive bowel sounds, mild tenderness at epigastrium, and generalized hypertympanic abdomen without hepatosplenomegaly.

**Figure 1.**
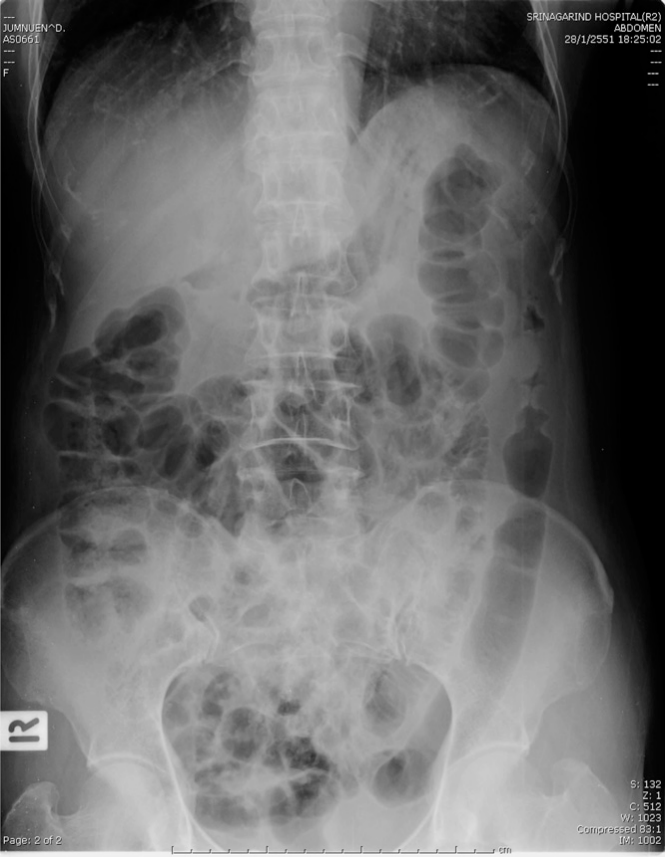
Plain abdomen showed diffuse small- and large-bowel dilatations.

Laboratory investigations showed eosinophilia (8.8%) and generalized small- and large-bowel dilatations (Figure 1). Liver function test, long gastrointestinal study, and ultrasonography of abdomen were all normal. She was treated with proton pump inhibitor with no improvement.

**Figure 2.**
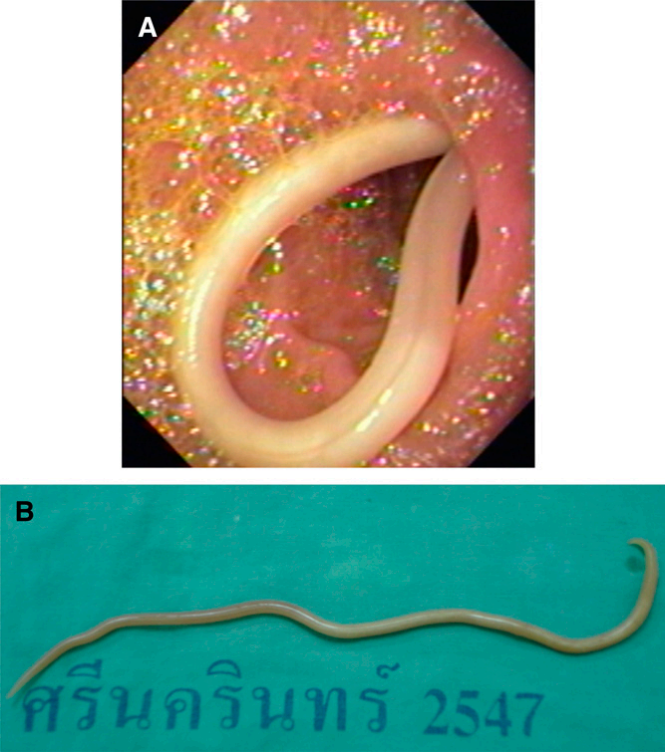
Ascaris lumbricoides in (**A**) the duodenum and (**B**) after removal. This figure appears in color at www.ajtmh.org.

The esophagogastroduodenoscopy showed severe gastritis with a smooth, creamy-colored, motile worm at the duodenal bulb (Figure 2). The worm was removed by endoscopic snare and identified as *Ascaris lumbricoides*; 400 mg albendazole was given orally.

**Figure 3.**
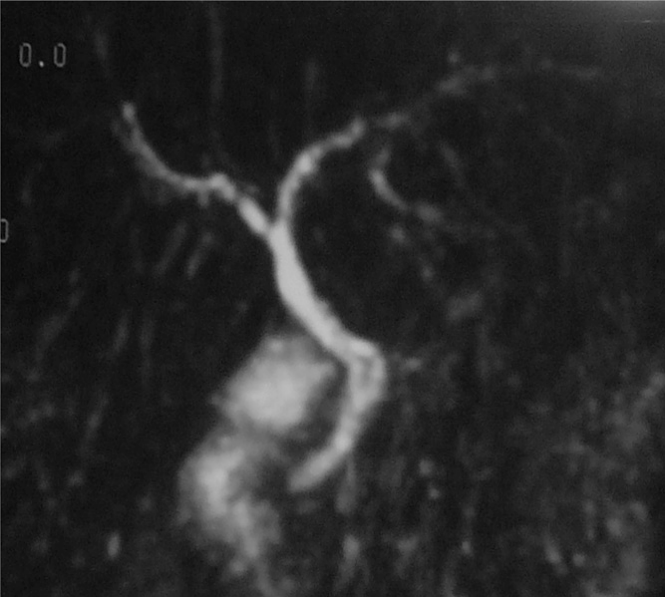
The MRCP showed a thin tubular filling defected with moderately dilatation in common bile duct.

At her 2-month follow-up visit, she still had a bloating sensation in her upper abdomen. The magnetic resonance cholangiopancreatography (MRCP) was performed and showed a thin tubular filling defected with moderate dilatation of the common bile duct (Figure 3). She refused an endoscopic retrograde cholangiopancreatography (ERCP), but she was successfully treated with albendazole (400 mg) for 7 days. At her 1-month follow-up visit, the stool examination was negative.

*A. lumbricoides* may live in the human jejunum for 1–2 years without causing any symptoms. After they migrate to other parts of the digestive system such as the duodenum, hepatobiliary tract, or pancreas, significant symptoms occur.^1^ In our patient, severe vomiting and abdominal pain developed when the worm migrated to the duodenum, an uncommon living site for this parasite. After worm removal and albendazole treatment, she still experienced abdominal discomfort in her epigastric area. Hepatobiliary ascariasis was shown by MRCP. Post-cholecystectomy has been shown to be a risk for developing hepatobiliary ascariasis.^2^ A single dose of albendazole therapy might aggravate another ascarid to migrate to the biliary tract through the Ampulla of Vater. Although ERCP can be both a diagnostic and therapeutic tool for hepatobiliary ascariasis,^1^,^2^ a 1-week course of albendazole was shown here to be effective.
